# Efficacy and Safety of Intensified vs Standard Prophylactic Anticoagulation Therapy in Patients Hospitalized With Coronavirus Disease 2019: Updated Systematic Review and Meta-analysis

**DOI:** 10.1093/ofid/ofad506

**Published:** 2023-10-10

**Authors:** Thomas C Scheier, Stephanie Carlin, Nicola K Wills, Sean Wasserman, Dominik Mertz, John W Eikelboom

**Affiliations:** Population Health Research Institute, Hamilton, Ontario, Canada; Department of Medicine, McMaster University, Hamilton, Ontario, Canada; Department of Medicine, University of Cape Town, Cape Town, South Africa; Department of Medicine, University of Cape Town, Cape Town, South Africa; Wellcome Centre for Infectious Diseases Research in Africa, Institute of Infectious Disease and Molecular Medicine, University of Cape Town, Cape Town, South Africa; Population Health Research Institute, Hamilton, Ontario, Canada; Department of Medicine, McMaster University, Hamilton, Ontario, Canada; Department of Health Research Methodology, Evidence, and Impact, Faculty of Health Sciences, McMaster University, Hamilton, Ontario, Canada; Population Health Research Institute, Hamilton, Ontario, Canada; Department of Medicine, McMaster University, Hamilton, Ontario, Canada

To  The  Editor—Multiple additional studies have been reported since the 2022 publication by members of our group of a systematic review and meta-analysis of randomized trials of intensified vs standard prophylactic anticoagulation therapy in patients hospitalized with coronavirus disease 2019 (COVID-19) [1]. Including all available data in an updated meta-analysis can be expected to provide more reliable and precise estimates of the effects of treatment on clinical outcomes and thereby strengthen guidance on the use of anticoagulation in patients hospitalized with COVID-19.

This updated systematic review and meta-analysis combine data from previously included randomized trials of inpatients with data from randomized trials identified by an updated search of PubMed and the Cochrane Central Register of Controlled Trials from 19 January 2022 (the end date of the previous search) to 6 July 2023 [1]. Two authors (T. C. S., S. C.) independently performed the search using the same search strategy previously reported, screened studies, extracted data, and resolved any disagreements through discussion. The main outcomes were all-cause mortality, major bleeding, and venous thromboembolism (VTE) at 30 days or at end of study follow-up, if this occurred earlier. Data were pooled by a random-effects model and are reported as risk ratios (RRs) and 95% CIs. Between-study heterogeneity was quantified with the *I*^2^ statistic and Cochran *Q* test [2]. A priori subgroup analyses examined treatment effects according to illness severity, as defined by need for intensive care unit (ICU) admission at study entry. If studies did not report outcomes separately for ICU and non-ICU cases, we categorized trials as ICU when >50% of randomized participants were admitted to the ICU at baseline. Publication bias was assessed by funnel plots for each reported outcome, and the robustness of the all-cause mortality outcome was explored per the “leave one out” approach. Analyses were performed with the meta package [3] and R software [4].

We identified an additional four studies [5–8], including 4305 patients, that met the eligibility criteria (Supplementary Figure 1*A*). When combined with the 10 inpatient studies in our previous report, our updated meta-analysis included 14 studies involving 9900 patients: 2703 ICU and 7197 non-ICU. All studies reported mortality and bleeding outcomes, and 12 reported VTE.

When compared with standard-dose prophylactic anticoagulation, intensified therapy was associated with a similar risk of mortality (RR, 0.89; 95% CI, .77–1.03) but reduced VTE by almost one-half (RR, 0.56; 95% CI, .42–.74). Risk of major bleeding was increased with the use of intensified anticoagulation (RR, 1.83; 95% CI, 1.30–2.57; Figure 1). Results were consistent across ICU and non-ICU settings. Leave-one-out sensitivity analysis showed consistent results for mortality. Funnel plots provided no evidence for important publication bias [9] (Supplementary Figure 1*B–D*).

**Figure 1.**
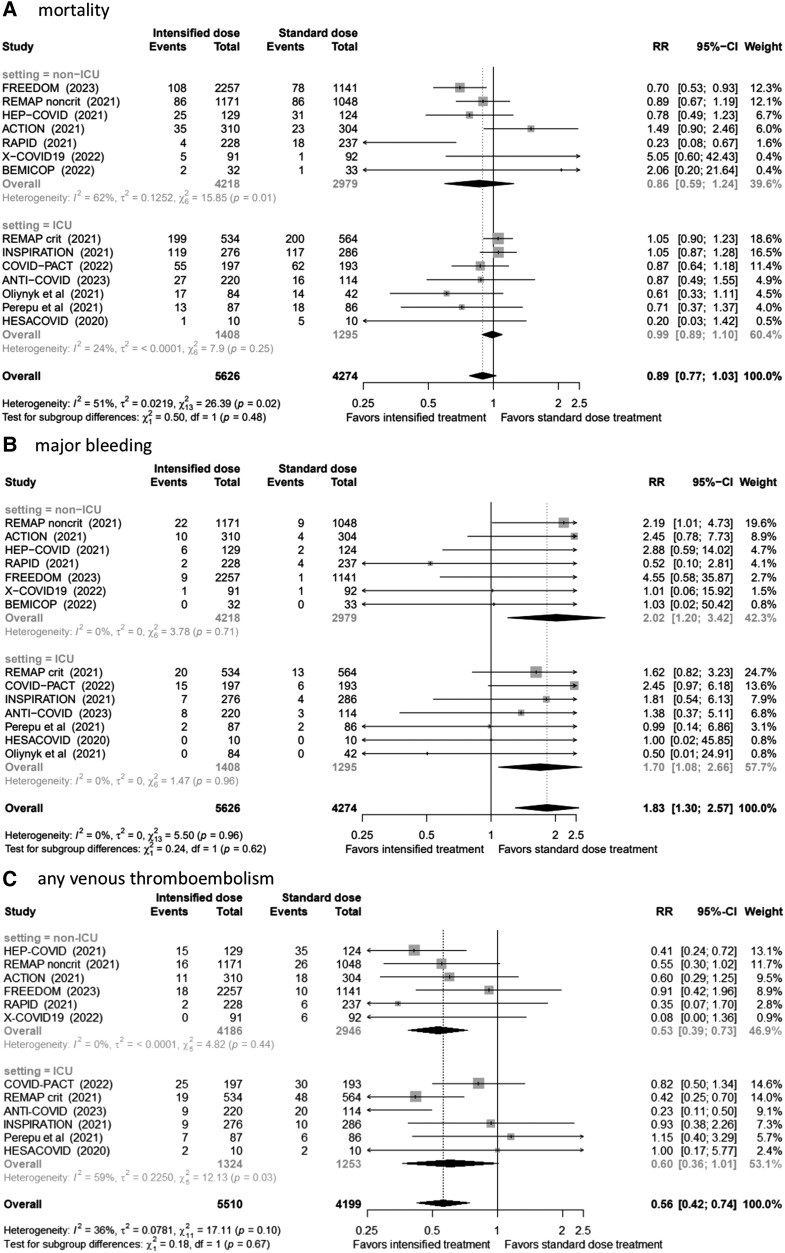
Forest plot: *A*, mortality; *B*, major bleeding; *C*, any venous thromboembolism, including overall and subgroup estimates. RR, risk ratio.

Strengths of this work are the inclusion of all available published data from randomized trials and the rigorous conduct of our analyses. Furthermore, although not formally evaluated, the apparent consistency of the results of different anticoagulation agents suggests that the results are generalizable irrespective of type of anticoagulant. Our analysis also has limitations, including various sources of heterogeneity that may influence interpretation of the pooled results. One study included different doses of anticoagulant in the intensified treatment group [6]. Two studies included asymptomatic deep vein thrombosis detected by screening in the VTE outcome [5, 7], and some studies did not report outcomes separately according to whether patients were in the ICU at study entry. The studies used several bleeding definitions, which may not be directly comparable. In addition, there was moderate heterogeneity for the mortality outcome, which appeared to be driven by an unexplained qualitative treatment interaction in non-ICU trials. Finally, most studies in our analyses were performed during earlier stages of the COVID-19 pandemic; therefore, the results may not be generalizable to patients affected by more recent variants of the virus (eg, omicron), among populations that have gained immunity from vaccines and infection, and in those receiving contemporary treatments for COVID-19.

In conclusion, our updated systematic review and meta-analysis demonstrate that in patients hospitalized with COVID-19, intensified vs standard-dose prophylactic anticoagulation reduces VTE at the cost of increased bleeding with no overall mortality benefit. These results are consistent with our previous report and likely provide the most reliable and precise estimates of treatment effects of intensified vs standard prophylactic anticoagulation on the outcomes of mortality, VTE, and bleeding.

## Supplementary Material

ofad506_Supplementary_Data

## Supplementary Data

Supplementary materials are available at *Open Forum Infectious Diseases* online. Consisting of data provided by the authors to benefit the reader, the posted materials are not copyedited and are the sole responsibility of the authors, so questions or comments should be addressed to the corresponding author.

## Notes

***Patient consent statement*.** The study does not include factors necessitating patient consent.

***Financial support.*** S. C. received speaker fees from BMS/Pfizer and Leo Pharma and advisory board fees from AstraZeneca, and Servier. S. W. is supported by the National Institutes of Health (K43TW011421 and U01AI170426). J. W. E. received honoraria and/or grants from Anthos, Bayer, BI, BMS, Daiichi-Sankyo, Ionis, Janssen, Merck, Pfizer, and USV.
