# Supplementary figures and images for: Efficacy and Safety of Intensified vs Standard Prophylactic Anticoagulation Therapy in Patients Hospitalized With Coronavirus Disease 2019: Updated Systematic Review and Meta-analysis
Source: Open Forum Infect Dis. 2023 Oct 10;10(11):ofad506. doi: 10.1093/ofid/ofad506 (PMC10633781; doi:10.1093/ofid/ofad506)

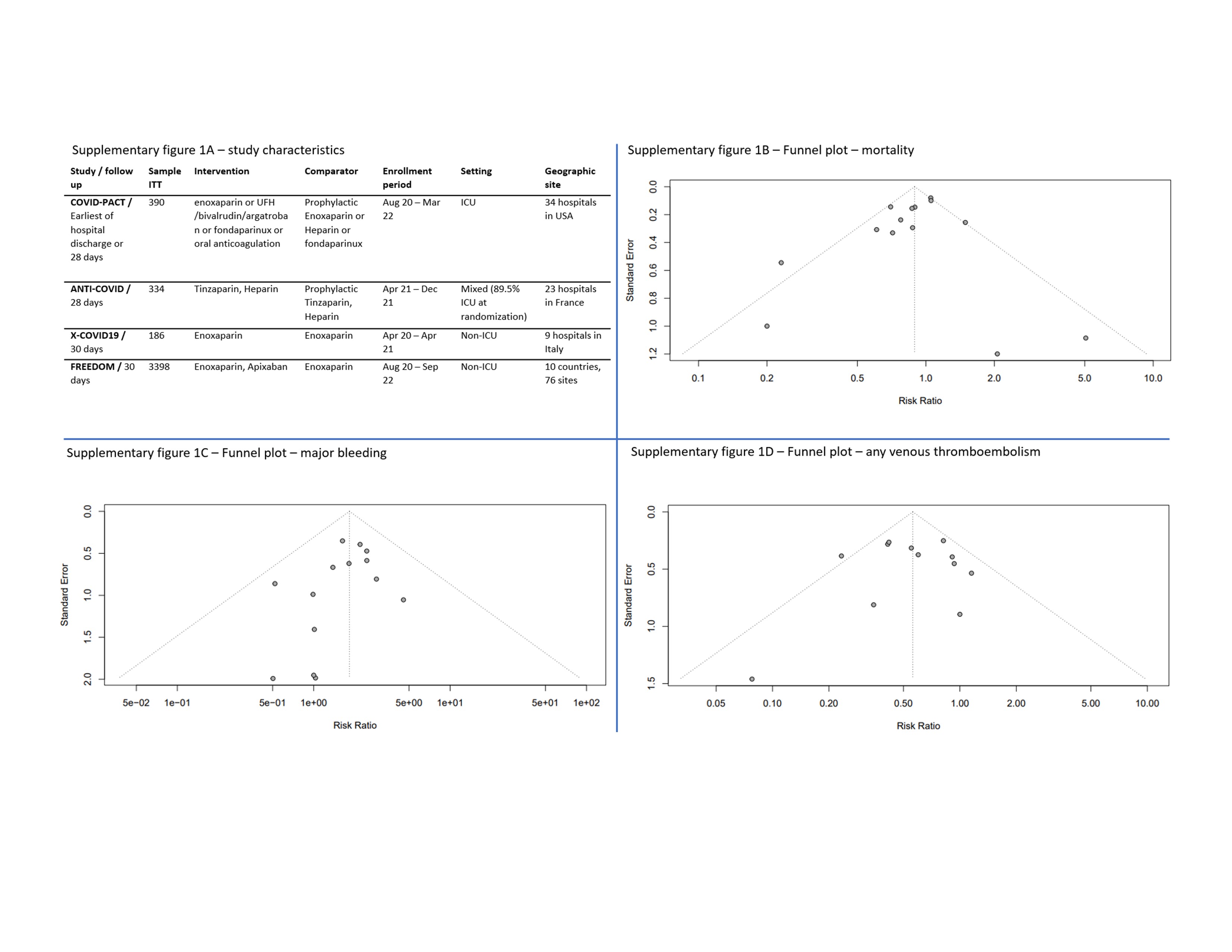

Supplement: ofad506_Supplementary_Data [file ofad506_supplementary_data.jpeg]
